# Impact of carotid atherosclerosis detection on physician and patient behavior in the management of type 2 diabetes mellitus: a prospective, observational, multicenter study

**DOI:** 10.1186/s12872-016-0401-5

**Published:** 2016-11-14

**Authors:** In-Kyung Jeong, Sin-Gon Kim, Dong Hyeok Cho, Chong Hwa Kim, Chul Sik Kim, Won-Young Lee, Kyu-Chang Won, Doo-Man Kim

**Affiliations:** 1 Department of Endocrinology and Metabolism, Kyung Hee University School of Medicine, Kyung Hee University Hospital at Gangdong, Seoul, South Korea; 2 Korea University Anam Hospital, Seoul, South Korea; 3 Chonnam National University Hospital, Gwangju, South Korea; 4 Sejong General Hospital, Gyeonggi-do, South Korea; 5 Hallym University Sacred Heart Hospital, Gyeonggi-do, South Korea; 6 Kangbuk Samsung Hospital, Seoul, South Korea; 7 Yeungnam University Medical Center, Daegu, South Korea; 8 Department of Internal Medicine, Kangdong Sacred Heart Hospital, Hallym University Medical Center, Gil-Dong, Gangdong-Gu, Seoul, South Korea

**Keywords:** Behavior, Cardiovascular disease, Carotid atherosclerosis, Diabetes mellitus, Type 2

## Abstract

**Background:**

This study compared carotid ultrasound (CUS) and traditional risk calculations in determining cardiovascular disease (CVD) risk in patients with type 2 diabetes mellitus (DM) and investigated whether awareness of CVD affects patient and/or physician behavior.

**Methods:**

In this prospective, observational, multicenter study, 797 participants with type 2 diabetes were assessed using CUS, the United Kingdom Prospective Diabetes Study Risk Engine (UKPDSRE) calculator, and the Framingham Risk Score (FRS) algorithm. Health-related behaviors and physician treatments were compared at baseline and at 6 months after assessment.

**Results:**

According to CUS, 43.5 % of the participants were at high risk (compared to 10.6 % and 4.3 % using the UKPDSRE and FRS approaches, respectively). Interestingly, 31.5 % of the patients with low risk scores according to the UKPDSRE calculator and 35.8 % of the patients with low risk scores according to the FRS algorithm were found to be at high risk according to CUS. The proportion of patients who achieved target LDL-C levels significantly increased after CUS. Moreover, increased awareness of atherosclerosis through CUS findings significantly altered physician treatment patterns and patient health-related behaviors.

**Conclusions:**

Carotid atherosclerosis was detected in more than 30 % of all participants with low or intermediate risk stratification scores. Improved awareness of atherosclerosis through CUS findings had a positive impact on both patient and physician behavior, resulting in improved CV risk management.

**Electronic supplementary material:**

The online version of this article (doi:10.1186/s12872-016-0401-5) contains supplementary material, which is available to authorized users.

## Background

Cardiovascular disease (CVD) is a major cause of mortality and morbidity in patients with type 2 diabetes mellitus (DM), making early diagnosis and treatment of atherosclerosis extremely important [1]. However, most patients with diabetes with subclinical atherosclerosis are asymptomatic [2]. In addition, the prevalence of silent myocardial ischemia (MI) is much higher in patients with diabetes compared to the general population [3]. Thus, in order to provide optimal medical therapy to prevent future cardiac events, identification of patients who are at high risk for CVD is of prime importance. The current guidelines on CVD prevention recommend targeted management of CV risk factors after assessment using one of the many available methods, even in asymptomatic patients.

Cardiovascular disease risk analysis can be performed using well-known risk-stratification approaches, including the Framingham Risk Score (FRS) algorithm [4] and the United Kingdom Prospective Diabetes Study Risk Engine (UKPDSRE) calculator [5]. The results of the FRS and UKPDSRE approaches, which include traditional CV risk factors, generally correlate with coronary heart disease risk [4]. However, a substantial number of people with low (<10 %) to intermediate (10–20 %) FRS scores go on to develop atherosclerosis [6]. Previous reports have also demonstrated that the UKPDSRE lacks adequate sensitivity and specificity for detection of subclinical atherosclerosis [7]. Therefore, additional tools are needed to improve CV risk assessment.

Recent investigations have shown that noninvasive techniques, such as carotid intima media thickness (CIMT), presence of plaque, coronary artery calcium score (CACS), ankle-brachial index (ABI), and aortic pulse wave velocity may accurately detect subclinical atherosclerosis that is associated with the development of cardiovascular or cerebrovascular diseases [8]. These studies have shown that imaging modalities are the best method for detecting the presence and extent of atherosclerosis. As such, it is important to conduct imaging studies in all patients regardless of the presence of traditional CV risk factors, such as hypertension, dyslipidemia, and diabetes mellitus, in order to comprehensively identify patients who are at risk for developing CVD. We chose to focus our study on CIMT because CUS is feasible in all individuals, dose not involve exposure to radiation, and is relatively inexpensive. When using imaging studies, a CACS >0, stenosis >50th percentile, or the presence of plaque are considered to be positive findings. These findings suggest a high risk of developing CVD according to the guidelines from the Screening for Heart Attack Prevention and Education (SHAPE), published by the Association for Eradication of Heart Attack (AEHA) [9].

However, the outcomes of this guideline have not yet been compared to those of the traditional guideline. Therefore, we analyzed the prevalences of abnormal carotid ultrasound (CUS) findings and compared them to traditional risk stratification results obtained using the FRS and UKPDSRE approaches.

Although physicians provide comprehensive treatment for diabetes, hypertension, and dyslipidemia, patient drug compliance is critical for optimal outcomes. If a physician assesses CVD risk and uses these results to educate the patient about ways to prevent CVD, the patient may implement lifestyle modifications or improve his/her drug compliance. However, no studies have yet investigated the effect of assessing subclinical atherosclerosis on patient behavior, or whether awareness of subclinical atherosclerosis alters physician treatment patterns.

Here we explored how two distinct assessment methods varied in their estimation of CV risk, a non-invasive imaging test (CUS) and traditional risk calculators (UKPDSRE, FRS). We also examined how awareness of being at high risk for CVD affected physician treatment patterns as well as patient behavior with respect to risk management. Our hypothesis was that receiving an explanation of CUS results, along with proper education about mitigating risk factors, would have a favorable effect on patient behavior and physician treatment plans.

## Methods

This prospective, observational, multicenter study included 797 patients with type 2 DM aged >40 years who had never undergone a carotid ultrasound examination. Participants were recruited from 24 hospitals in Korea. We excluded patients who had previously undergone carotid artery ultrasound, or who had a history of coronary artery disease, symptomatic congestive heart failure, coronary revascularization, cerebrovascular disease, stroke, transient ischemic attack, or documented peripheral vascular disease (e.g. peripheral artery disease, abdominal aneurysm, or carotid artery stenosis). The investigation protocol was approved by the institutional review boards of each institution involved in the study. After obtaining informed written consent, the height, weight, and body mass index (BMI) (weight/height^2^, kg/m^2^) of each patient were measured. Blood pressure was measured using a standard mercury sphygmomanometer. All patients were interviewed prior to CUS examination. Questionnaires were administered using one-on-one interviews and self-reporting techniques to collect data on smoking; alcohol use; stress; dietary habits; physical activity; past history of hypertension, dyslipidemia, and atrial fibrillation; medication compliance; and family history of CVD. The validated Korean version [10] of Morsky’s self-reported questionnaire [11] was used to assess medication compliance. Levels of fasting plasma glucose, HbA_1C_, total cholesterol (TC), triglyceride (TG), high-density lipoprotein cholesterol (HDL-C), and low-density lipoprotein cholesterol (LDL-C); current medications; and the microalbumin-to-creatinine ratio within the past 1 month were collected by reviewing patient medical records.

All subjects were assessed by CUS. Carotid intimal-media thickness (IMT) was measured with the patient in the supine position. A high-resolution B-mode ultrasound machine with a 7.5-MHz transducer was used on the bilateral segments of the carotid arteries. The carotid IMT was measured on the posterior far wall of the left carotid artery. At least 4 measurements were taken, each about 1 cm proximal to the bifurcation. Positive criteria for carotid atherosclerosis were defined as ≥1 mm of intima medial thickness or the presence of plaque.

Although CUS was performed separately in the 24 different hospitals, each used a standardized protocol recommended by the Mannheim carotid IMT consensus report [12]. In addition, to adjust for potential intercenter variations due to different sonographers, every hospital used Intimascope software (Media Cross Co, Ltd, Tokyo, Japan) for measurement. This software performs automated IMT measurements based on an algorithm that delineates the lumen-intima and media adventitial interfaces [13].

Patients were stratified by risk using the UKPDSRE and FRS assessments. A total of 622 patients provided all required information to be assessed by the UKPDSRE calculator and 648 patients provided sufficient information to be assessed by the FRS algorithm. A total of 622 patients were assessed by CUS, UKPDSRE, and FRS. The UKPDS calculator classified subjects into low (<15 %), intermediate (15–30 %), or high (>30 %) 10-year risk levels for CVD based on age, sex, duration of diabetes, smoking, systolic blood pressure, total cholesterol, HDL, ethnicity, and HbA_1C_ [4]. The FRS algorithm categorized subjects into low (<10 %), intermediate (10–19 %), or high (≤20 %) 10-year risk levels for symptomatic CVD according to age, sex, lipid levels, blood pressure, smoking, and presence of diabetes [5].

Blood samples were collected six months after carotid IMT assessment to measure levels of TC, HDL-C, TG, and LDL-C. Patients were also re-examined for changes in responses to interview questions, physician prescriptions, and patient behaviors.

All statistical analyses were completed using SAS (version 9.2, USA). All data are presented as means ± standard deviations (SDs) or as numbers (percentages). To compare clinical characteristics between the two groups, an independent *t*-test was used for continuous variables and a chi-squared test was used for categorical variables. Multiple logistic regression analysis was used to analyze the association between carotid IMT and CVD risk factors. A paired *t*-test was used to measure changes in patient behavior before and after they were informed about their subclinical carotid atherosclerosis risk. Differences with a p-level <0.05 were considered statistically significant.

## Results

### Baseline characteristics of the subjects with type 2 DM

Table 1 summarizes the clinical and laboratory measurements of the subjects with type 2 DM included in this study. The mean patient age was 60 years and the mean BMI was 25.1 ± 3.1 kg/m^2^. Half of the subjects had a >10-year duration since diagnosis with type 2 DM. According to patient questionnaires, the most frequent co-morbidities were hypertension (50.69 %) and dyslipidemia (37.5 %) (not shown in Table 1). Examination of medical records revealed that antihypertensive drugs, statins, and antiplatelet agents were prescribed to 43.3 %, 42.3 %, and 41.2 % of all patients, respectively. Approximately 20 % of the patients were current smokers. The mean HbA_1C_ level was 60 ± 18.6 mmol/mol. The mean LDL-C level was 2.57 ± 0.86 mmol/L.

**Table 1:** Baseline clinical characteristics of patients with versus without subclinical atherosclerosis

		Atherosclerosis Findings via Carotid IMT		
	Total (*N* = 797)	Negative (*N* = 455)	Positive (*N* = 342)	^*^ *P*-value
Age (years)	60.0 ± 9.5	57.9 ± 9.2	63.5 ± 9.0	<0.001
Sex (percent male)	395 (49.6)	227 (49.9)	175 (51.2)	0.721
BMI (kg/m^2^)	25.1 ± 3.1	25.3 ± 3.4	24.9 ± 3.0	0.067
Waist circumference (cm)	87.2 ± 8.2	87.6 ± 8.6	86.7 ± 7.6	0.164
Blood pressure (mmHg)				
Systolic	125.3 ± 14.5	124.5 ± 14.7	126.5 ± 14.2	0.060
Diastolic	75.3 ± 10.1	75.7 ± 9.6	74.7 ± 10.6	0.170
DM duration (years)	8.1 ± 7.1	7.4 ± 6.7	9.0 ± 7.6	0.002
Medication use [N (%)]				
Antihypertensive drug	345 (43.3)	174 (38.2)	171 (50.0)	0.001
Statin	337 (42.3)	195 (42.9)	142 (41.5)	0.705
Antiplatelet agent	328 (41.2)	170 (37.4)	158 (46.2)	0.012
Current smoker (%)	153 (19.2)	81 (17.9)	72 (21.1)	0.253
Glucose (mmol/L)	8.1 ± 2.8	8.0 ± 2.7	8.2 ± 3.0	0.591
HbA1C (mmol/mol)	60 ± 18.6	60 ± 17.5	61 ± 19.7	0.417
Log hs-CRP (mg/L)	−1.2 ± 1.8	−1.4 ± 1.7	−1.0 ± 1.8	0.017
Total cholesterol (mmol/L)	4.4 ± 1.0	4.4 ± 1.0	4.5 ± 1.0	0.710
Triglycerides (mmol/L)	1.7 ± 1.1	1.7 ± 1.2	1.7 ± 1.0	0.884
LDL-C (mmol/L)	2.6 ± 0.9	2.5 ± 0.9	2.6 ± 0.8	0.284
HDL-C (mmol/L)	1.2 ± 0.4	1.3 ± 0.3	1.2 ± 0.4	0.594
UKPDS risk engine score	16.8 ± 12.1	13.3 ± 8.7	20.4 ± 13.6	<0.001
UKPDS risk engine (%) (*N* = 622)				
High	66 (10.6)	15 (4.3)	51 (18.8)	
Intermediate	213 (34.2)	101 (28.8)	112 (41.3)	<0.001
Low	343 (55.2)	235 (67.0)	108 (39.9)	
Framingham risk score	7.7 ± 6.4	6.2 ± 5.7	9.20 ± 7.1	<0.001
Framingham risk engine (%) (*N* = 648)				
High	28 (4.3)	6 (1.6)	22 (7.8)	
Intermediate	195 (30.1)	88 (24.0)	107 (38.1)	<0.001
Low	425 (65.6)	273 (74.4)	152 (54.1)	

*IMT* intima medial thickness, *BMI* body mass index, *DM* diabetes mellitus, *CRP* C-reactive protein, *LDL-C* low-density lipoprotein cholesterol, *HDL* high-density lipoprotein cholesterol, *UKPDS* United Kingdom Prospective Diabetes Study

^*^ *P*-value: comparison of clinical data between patients who were negative versus positive for carotid atherosclerosis according to carotid IMT

### Estimated cardiovascular risk of the subjects

In total, 42.9 % of the subjects with diabetes had a positive finding for atherosclerosis according to carotid US (Table 1). Of the 622 patients assessed using the UKPDS calculator, 43.6 % were positive for atherosclerosis according to carotid US. The UKPDS risk calculator determined that 343 (55.2 %) patients were at low risk for CVD, 213 (34.2 %) patients were at intermediate risk for CVD, and 66 (10.6 %) patients were at high risk for CVD. The FRS algorithm determined that 425 (65.6 %) patients were at low risk for CVD, 195 (30.1 %) patients were at intermediate risk for CVD, and 28 (4.3 %) patients were at high risk for CVD (Table 1). The 10-year risk of CVD was higher in the UKPDS high-risk group compared to the FRS high-risk group (10.6 % vs. 4.3 %, *p* < 0.0001).

We also calculated the UKPDS and FRS cutoff points for the prediction of positive CUS (IMT > 1 mm). The UKPDS cutoff was 14.52 (sensitivity 66.38 %, specificity 61.99 %) and the FRS cutoff was 14 (sensitivity 72.75 %, specificity 46.62 %).

There was a significant correlation between CV risk score and carotid IMT. The correlation coefficient between UKPDS score and mean IMT was 0.295 (*p* < 0.001). The correlation coefficient between FRS score and mean IMT was 0.227 (*p* < 0.001).

We next investigated whether the UKPDSRE or FRS calculator is a superior predictor of positive CUS. The area under the UKPDSRE ROC curve was 0.677 (95 % CI, 0.635, 0.719), while the area under the FRS ROC curve was 0.629 (95 % CI, 0.584, 0.672); this difference was significant (*P* = 0.001). Therefore, the UKPDSRE calculator was better at predicting positive CUS than the FRS algorithm.

### Patient clinical characteristics according to positive carotid ultrasound findings

Patients with positive carotid ultrasound findings were significantly older (63.5 ± 9.0 vs. 57.9 ± 9.2 years), had a longer duration of diabetes (9.0 ± 7.6 vs. 7.4 ± 6.7 years), used more antihypertensive medication (50.0 vs. 38.2 %) and antiplatelet agents (46.2 vs. 37.4 %), and had higher log hs-CRP levels (−1.4 ± 1.7 vs. −1.0 ± 1.8 mg/L) compared to subjects with negative carotid US findings (Table 1). Furthermore, the UKPDSRE and FRS scores were higher in subjects with positive carotid IMT findings. In addition, the percentages of high risk patients as classified by the UKPDSRE and FRS systems were higher for subjects with positive carotid IMT findings compared to subjects with negative findings (18.8 vs. 4.3 % and 7.8 vs. 1.7 %, respectively) (Table 1).

Multiple logistic regression analysis was next performed to investigate the associations between CV risk factors and abnormal carotid US findings after adjusting for age and sex. Carotid IMT was positively correlated with LDL-C level. There was a trend towards a positive association between ex- and current smokers and positive carotid IMT findings. No significant associations were noted between positive CUS findings and BP, BMI, DM duration, HbA_1C_, UKPDSRE score, or FRS score (Table 2).

**Table 2:** Associations between carotid intima medial thickness and cardiovascular disease risk factors^a^

	OR	95 % CI	*P*-value
BMI	1.051	0.945, 1.169	0.835
Systolic blood pressure	1.010	0.994, 1.027	0.219
DM duration	0.997	0.963, 1.033	0.887
Ex & current smokers	1.771	0.907, 3.459	0.094
HbA_1C_	1.006	0.843, 1.201	0.946
Triglycerides	1.002	0.999, 1.005	0.142
LDL-C	1.018	1.003, 1.032	0.017
HDL-C	1.013	0.985, 1.042	0.360
UKPDS risk engine score	27.003	0.057, 999.999	0.294
Framingham risk score	0.971	0.900, 1.047	0.441

*OR* odds ratio, *CI* confidence interval, *BMI* body mass index, *DM* diabetes mellitus, *LDL-C* low-density lipoprotein cholesterol, *HDL* high-density lipoprotein cholesterol, *UKPDS* United Kingdom Prospective Diabetes Study

^a^Values adjusted for age and sex

### Prevalence of carotid atherosclerosis according to risk stratification scores

**Fig. 1:**
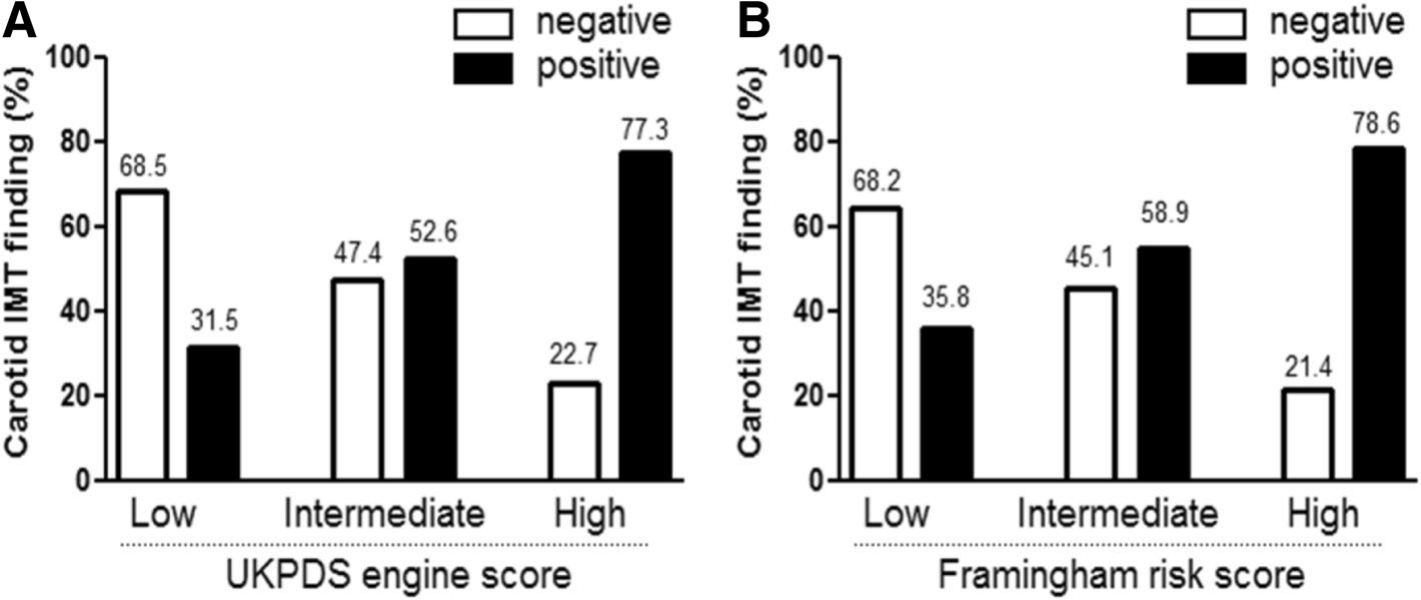
Prevalences of carotid atherosclerosis according to UKPDS engine score (**a**) and Framingham risk score (**b**). Open bar: negative findings from carotid ultrasound. Black bar: positive findings from carotid ultrasound

According to the UKPDS risk engine, the prevalences of positive carotid US findings were 31.5 % in the low-risk group, 52.6 % in the intermediate-risk group, and 77.3 % in the high-risk group. According to the FRS algorithm, the prevalences of abnormal carotid US findings were 35.8 % in the low-risk group, 54.9 % in the intermediate-risk group, and 78.6 % in the high-risk group (Fig. 1). Overall, about one-third of the patients in both low-risk groups had a positive finding according to carotid US. On the other hand, only about 20 % of the patients in both high-risk groups had a negative finding according to carotid US (Fig. 1).

### Factors contributing to the discrepancy between UKPDS risk engine stratification and carotid US findings

In total, 39.9 % of subjects with a positive CUS finding were classified as low-risk according to the UKPDSRE calculator (Table 1). To identify the factors that were associated with abnormal CUS findings in patients assessed by the UKPDSRE calculator, we compared the clinical parameters of patients with negative versus positive CUS findings in each risk-stratified group. In the low-risk group, patients with positive CUS findings were significantly older and had a significantly lower waist circumference, lower diastolic BP, a higher prevalence of hypertension and dyslipidemia, and a higher prevalence of antihypertensive medication use compared to subjects with negative CUS findings. In the intermediate-risk group, positive CUS findings were associated with increased age, female sex, and higher hs-CRP levels compared to subjects with negative CUS findings. In the high-risk group, no significant associations were observed between any parameter and positive CUS findings between the two groups (Additional file 1: Table S1). This finding may be due to the relatively small number of subjects in the high-risk group.

### Changes in treatment patterns after CUS measurements

Changes in physician prescriptions were investigated at 6 months after the initial CUS measurements. Awareness of high-risk CUS findings significantly altered physician treatment patterns (*p* = 0.011) for managing major CV risk factors. In addition, significant increases in the addition and dosages of anti-hypertensive drugs (*p* = 0.013) and antiplatelet agents (*p* = 0.003) were observed in patients with positive CUS findings (Table 3).

**Table 3:** Changes in treatment patterns after knowledge of subclinical carotid atherosclerosis results

	Carotid Artery Ultrasound Findings		
	Negative, N (%) *N* = 455	Positive, N (%) *N* = 342	*P*-value
Treatment pattern			
Changed	96 (24.7)	107 (33.4)	0.011
Additional medications			
Anti-hypertensive drugs	32 (8.3)	45 (18.1)	0.013
Lipid-lowering drugs	52 (13.4)	44 (13.8)	0.893
Antiplatelet agents	26 (6.7)	43 (13.4)	0.003
Achievement of treatment target goals			
BP (<130/80 mmHg)			
At baseline	210 (49.1) (*N* = 428)	142 (46.3) (*N* = 307)	0.452
After 6 months	199 (46.5) (*N* = 428)	139 (45.3) (*N* = 307)	0.744
Baseline vs. 6 months	*P* = 0.389	*P* = 0.785	
LDL-C (<2.59 mmol/L)			
At baseline	112 (57.7) (*N* = 194)	80 (55.6) (*N* = 144)	0.690
After 6 months	130 (67.0) (*N* = 194)	105 (72.9) (*N* = 144)	0.243
Baseline vs. 6 months	*P* = 0.022	*P* < 0.001	
Calculated LDL^a^ (<2.59 mmol/L)			
At baseline	125 (56.8) (*N* = 220)	96 (56.8) (*N* = 169)	0.998
After 6 months	157 (71.4) (*N* = 220)	126 (74.6) (*N* = 169)	0.483
Baseline vs. 6 months	*P* = 0.022	*P* < 0.001	

*BP* blood pressure, *LDL-C* low-density lipoprotein cholesterol

^a^Calculated LDL = total cholesterol – (triglyceride/5) – HDL

The percentage of subjects who achieved the target LDL-C goal (<2.59 mmol/L) was significantly higher 6 months after CUS examination in patients with negative CUS findings and also in patients with positive CUS findings (Table 3). Abnormal CUS findings affected physician behavior regardless of patient risk level according to the UKPDSRE calculator. A significant change in treatment patterns for antihypertensive drug use (7.0 vs. 14.6 %) was observed in patients with positive CUS findings who were identified as low-risk according to the UKPDS calculator. A significant change in treatment patterns for antiplatelet agent use (0 vs. 22.0 %) was also observed in patients with positive CUS findings who were identified as high-risk according to the UKPDSRE calculator (Additional file 1: Table S2).

### Changes in patient behavior after education based on carotid US results

**Fig. 2:**
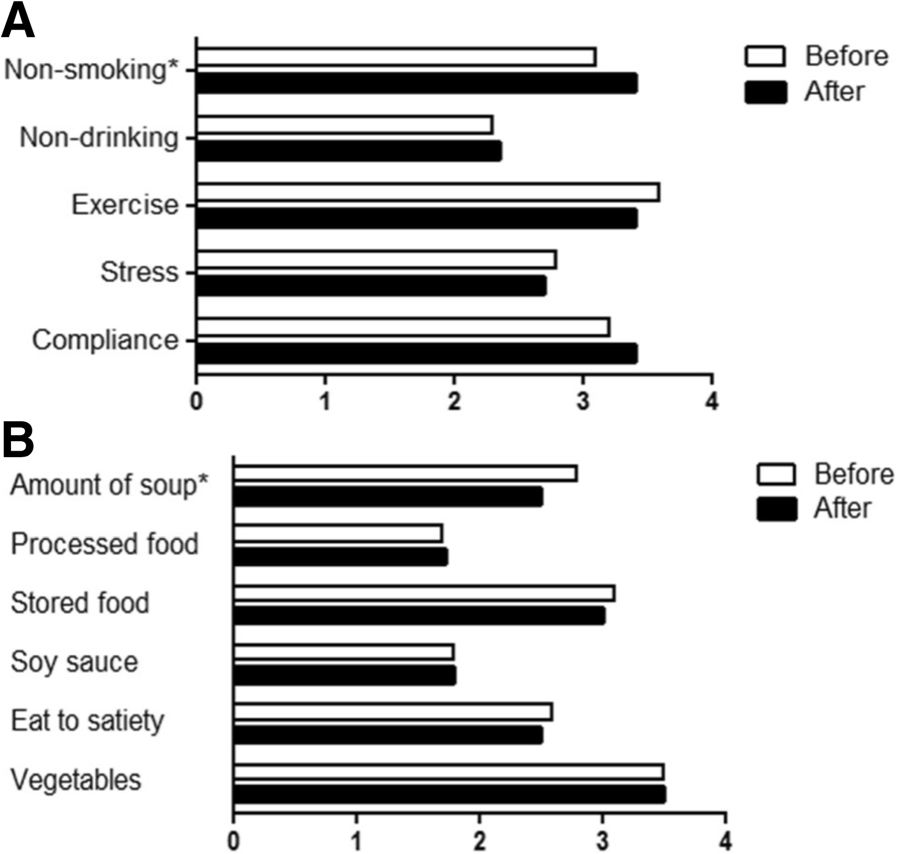
Changes in behavior (**a**) and diet (**b**) in patients after education based on carotid US results. [※: *p* < 0.05 between behavior before (open bar) and after (black bar) awareness of positive findings from carotid ultrasound]

Interviews were performed 6 months after patients were informed of their carotid US results. Overall, patients who were informed of their CUS results exhibited significant changes in their health-related behaviors. For example, the rates of smoking cessation and dietary changes (*p* < 0.005 each) were both increased at the 6-month follow-up visit. Moreover, the percentage of patients who had quit smoking had significantly increased and the amount of soup intake had reduced significantly at six months after the CUS examination (Fig. 2). This finding suggests that the patients tried to reduce their salt intake by decreasing their soup consumption.

## Discussion

Our results suggest that CUS can identify CVD-vulnerable patients out of the population of patients with type 2 DM with low-risk or intermediate-risk stratification scores. In addition, improved awareness of CVD risk based on carotid IMT results can improve CV risk management by increasing the prevention efforts of both physicians and patients.

According to CUS, 271 (43.5 %) patients were at high risk for CVD. In contrast, only 66 (10.6 %) and 28 (4.3 %) patients were at high risk for CVD according to the UKPDSRE calculator and the FRS algorithm, respectively. We also found that more high-risk patients were identified using the UKPDSRE calculator compared to the FRS algorithm (10.6 vs. 4.5 %, *p* < 0.0001). This finding was not surprising, since the UKPDS risk engine was developed especially for use with patients with diabetes [5]. As such, this method has a higher prognostic value for coronary heart disease in patients newly diagnosed with type 2 diabetes [14]. Also, the UKPDSRE calculator provided the highest odds ratios for predicting carotid atherosclerosis in Korean patients with type 2 diabetes compared to the FRS and the SCORE methods [15]. However, the prevalences of positive CUS findings were very similar in the low-risk (31.5 % vs. 35.8 %), intermediate-risk (52.6 % vs. 54.9 %), and high-risk (77.3 % vs. 78.6 %) groups for both the UKPDS risk engine and the FRS algorithm, respectively (Fig. 1).

The FRS and UKPDSRE approaches, both of which include traditional CV risk factors, have been validated for predicting CV risk in Asian populations [15–17]. Although the results of these approaches are generally correlated with subclinical atherosclerosis, the majority of CVD events occur in patients with low or intermediate risk of CVD [4]. To our surprise, one-third of the low-risk patients according to both the UKPDSRE and FRS classifications had a positive CUS finding. Of the patients classified as low-risk based on their UKPDSRE score, 39.9 % had a positive CUS finding. One previous study found that 32.8 % of all women and 40.5 % of all men with low (<10 %) to intermediate (10–20 %) risk of CVD according to the FRS algorithm had subclinical atherosclerosis [6]. Analysis of factors related to atherosclerosis in the low-risk group indicated that positive CUS findings were significantly associated with older age, higher prevalences of hypertension and dyslipidemia, and a higher prevalence of antihypertensive medication history.

Carotid IMT and the presence of carotid plaque are important markers of subclinical atherosclerosis that can be used to predict cardiovascular morbidity. Many epidemiology studies, such as the Atherosclerosis Risk in Communities (ARIC) study [18] and the Insulin Resistance Atherosclerosis Study (IRAS) [19], have demonstrated that age, male sex, smoking, hypertension, dyslipidemia, and postmenopausal status are independent correlates of carotid atherosclerosis. In particular, Chin et al. [20] showed that LDL-C levels in males and HDL-C levels in females were risk factors for IMT progression among patients newly diagnosed with type 2 diabetes. We found that old age, diabetes duration, percentage of antihypertensive medication use, antiplatelet agent use, and log hs-CRP level were significantly higher in patients with positive CUS findings. After adjustment for age and sex, LDL-C was an independent correlate of subclinical carotid atherosclerosis. These data suggest that LDL-C should be managed to protect or delay the progression of atherosclerosis. Therefore, comprehensive management of CV risk factors and patient adherence to the treatment plan may prevent or delay the progression of atherosclerosis.

We also assessed how patient lifestyle and physician prescriptions changed after receiving the CUS results, as well as how knowing these results affected the achievement of target lipid and BP levels after 6 months. Among the patients with positive CUS findings, significant increases in the use or dosage of anti-hypertensive drugs and antiplatelet agents were observed compared to those in patients with negative CUS findings. We speculate that learning that a patient had subclinical atherosclerosis of the carotid artery may have encouraged physicians to more intensively manage patient risk factors for atherosclerosis. In addition, this intervention was associated with a significant improvement in the achievement of target LDL-C levels, even though a change in medication prescriptions related to hypercholesterolemia was not observed. This finding implies that medication compliance for lipid-lowering drugs might increase after patients receive their CUS findings, regardless of whether these findings are negative or positive. Hong et al. [21] reported that among asymptomatic patients with hypertension, atherosclerosis detection by CUS significantly increased the proportion of patients who achieved their target LDL-C levels compared to patients who received a negative CUS finding. However, we found that patient knowledge of CUS findings improved outcomes, regardless of whether the results were positive or negative. As such, we propose that CUS is a beneficial tool for increasing adherence to lipid-lowering drug regimens as part of CV risk management in patients with type 2 diabetes.

Patient behaviors also changed significantly after the patients received their CUS results. Specifically, patients who underwent CUS examination subsequently reduced smoking and salt intake (for the latter, by reducing soup consumption). We infer that since consumption of Korean soup or stew has been shown to be associated with high salt intake [22], patients made an effort to reduce their salt intake by decreasing their soup consumption. Thus, our data indicate that knowledge of carotid US results and subsequent explanation of the relevant CVD risks is a useful approach for enabling patients to achieve the recommended lifestyle modifications. Furthermore, CUS is a very helpful tool that enables patients to better understand their atherosclerosis status, particularly when CUS imaging results are employed. Our results thus indicate that explanation of CUS results assists patients with diabetes and their physicians to achieve patient therapeutic targets through behavior changes and medication plan alterations.

This study did have some limitations. First, the study period was only 6 months, which is a relatively short period of time for full evaluation of CVD event outcomes. Second, although our results indicated that awareness of CUS results may positively influence physician management of CV risk factors and patient behavior, correlation does not imply causality. Third, we did not evaluate the quality or area of the carotid plaques found by CUS. Several studies have shown that the quality of plaque and the plaque area are more strongly predictive of CV events than IMT [23, 24]. Fourth, we defined a positive CUS finding as an IMT ≥ 1 mm or the presence of plaque. We chose these criteria based on several large clinical studies (e.g. the ARIC study and studies performed in Finland) that compared the hazard ratios between CIMT ≥ 1 mm and < l mm [25–27]. Specifically, the ARIC study showed that the hazard ratio comparing extreme mean CIMT (≥1 mm) to not extreme CIMT (<1 mm) was 5.07 for women and 1.85 for men. Above 1 mm, the CV event rates were elevated [26]. However, this cutoff point was derived from a non-Asian population. To more accurately predict CV risk in future studies, a more comprehensive investigation of the optimal CIMT cutoff point to predict CV risk in an Asian population would be beneficial. Finally, all subjects in the present study were Asian, and thus our findings may not be applicable to other populations.

## Conclusions

Our data indicate that CUS screening is an effective method for identifying patients with subclinical atherosclerosis, even among patients considered to be low-risk according to the UKPDRES or FRS approach. In addition, educating patients with type 2 DM about their atherosclerosis risk as determined by their CUS results may result in improved management of CV risk factors.

## Additional file

Additional file 1:
**Table S1.** Clinical characteristics of subjects according to subclinical carotid atherosclerosis and UKPDS risk engine score. **Table S2.** Changes in treatment patterns after knowledge of subclinical carotid atherosclerosis results according to UKPDS risk engine score. (DOCX 33 kb)

## Abbreviations

AEHA: Association for Eradication of Heart Attack
BMI: Body mass index
CUS: Carotid ultrasound
CVD: Cardiovascular disease
DM: Diabetes mellitus
FRS: Framingham risk score
HDL-C: High-density lipoprotein cholesterol
IMT: Intima media thickness
LDL-C: Low-density lipoprotein cholesterol
MI: Myocardial ischemia
SD: Standard deviation
SHAPE: Screening for heart attack prevention and education
TC: Total cholesterol
TG: Triglyceride
UKPDS: United Kingdom Prospective Diabetes Study Risk Engine
