# Supplementary material for: Impact of carotid atherosclerosis detection on physician and patient behavior in the management of type 2 diabetes mellitus: a prospective, observational, multicenter study
Source: BMC Cardiovasc Disord. 2016 Nov 14;16:220. doi: 10.1186/s12872-016-0401-5 (PMC5109726; doi:10.1186/s12872-016-0401-5)
Supplement: Additional file 1: — Table S1. Clinical characteristics of subjects according to subclinical carotid atherosclerosis and UKPDS risk engine score. Table S2. Changes in treatment patterns after knowledge of subclinical carotid atherosclerosis results according to UKPDS risk engine score. (DOCX 33 kb) [file 12872_2016_401_MOESM1_ESM.docx]

**Table S1**. Clinical characteristics of subjects according to subclinical carotid atherosclerosis and UKPDS risk engine score

|  | **United Kingdom Prospective Diabetes Study Risk Engine Score** | | | | | | | | |
| --- | --- | --- | --- | --- | --- | --- | --- | --- | --- |
|  | **Low** | | | **Intermediate** | | | **High** | | |
| **Carotid IMT** | **Negative** | **Positive** | **P-value** | **Negative** | **Positive** | **P-value** | **Negative** | **Positive** | **P-value** |
| Number of patients | 235 | 108 |  | 101 | 112 |  | 15 | 51 |  |
| Age (years) | 53.9±7.7 | 58.4±8.1 | <0.001 | 62.5±7.9 | 65.1±8.1 | 0.020 | 71.5±9.2 | 69.0±7.1 | 0.270 |
| Sex (% male) | 98 (41.7) | 41 (38.0) | 0.512 | 70 (69.3) | 61 (54.5) | 0.026 | 11 (73.3) | 42 (82.4) | 0.471 |
| BMI (kg/m^2^) | 25.5±3.5 | 25.2±2.7 | 0.373 | 25.2±3.0 | 25.0±3.3 | 0.586 | 25.8±3.6 | 24.8±2.5 | 0.305 |
| Waist circumference (cm) | 86.9±9.0 | 84.8±7.1 | 0.033 | 89.3±7.8 | 87.7±7.5 | 0.157 | 89.9±9.8 | 88.6±8.6 | 0.647 |
| Blood pressure (mmHg)  Systolic  Diastolic | 124.5±13.9  76.9±9.4 | 122.6±13.7  73.5±10.8 | 0.260  0.003 | 124.7±16.5  74.7±10.7 | 128.1±13.9  75.5±11.7 | 0.104  0.610 | 127.1±14.1  73.2±7.3 | 130.4±14.8  75.8±10.2 | 0.441  0.356 |
| DM duration (years) | 6.1±5.4 | 6.8±5.7 | 0.232 | 9.6±7.3 | 9.0±7.2 | 0.605 | 11.2±10.0 | 13.3±9.5 | 0.473 |
| Associated disease, N (%)  Hypertension  Dyslipidemia | 100 (42.6)  87 (37.0) | 63 (58.3)  54 (50.0) | 0.007  0.023 | 52 (51.5)  37 (36.6) | 69 (61.6)  45 (40.2) | 0.136  0.596 | 9 (60.0)  4 (26.7) | 28 (54.9)  21 (41.2) | 0.727  0.309 |
| Medication use  Antihypertensive  Statin  Antiplatelet | 82 (34.9)  104 (44.3)  90 (38.3) | 53 (49.1)  57 (52.8)  50 (46.3) | 0.013  0.142  0.162 | 43 (42.6)  45 (44.6)  42 (41.6) | 54 (48.2)  43 (38.4)  52 (46.4) | 0.409  0.362  0.477 | 8 (53.3)  6 (40.0)  6 (40.0) | 23 (45.1)  20 (39.2)  23 (45.1) | 0.574  0.956  0.727 |
| Current smokers, N (%) | 33 (14.0) | 9 (8.3) | 0.134 | 23 (22.8) | 34 (30.4) | 0.212 | 6 (40.0) | 22 (43.1) | 0.829 |
| Glucose (mmol/L)  HbA1c (mmol/mol) | 8.0±2.5  56±15.3 | 7.7±2.4  55±15.3 | 0.346  0.563 | 8.2±3.0  65±19.7 | 8.2±2.8  63±20.8 | 0.935  0.559 | 7.8±2.8  66±25.1 | 8.7±4.0  69±20.8 | 0.427  0.609 |
| hs-CRP (mg/L) | 1.2±2.5 | 1.6±3.7 | 0.491 | 0.4±0.7 | 1.9±4.2 | 0.020 | 0.8±0.7 | 0.9±1.1 | 0.704 |
| Total cholesterol (mmol/L) Triglycerides (mmol/L)  LDL-C (mmol/L)  HDL-C (mmol/L)  Apo B/A1 | 4.3±1.0  1.5±0.9  2.4±0.8  1.3±0.4  0.6±0.1 | 4.3±0.9  1.5±0.7  2.4±0.8  1.4±0.5  0.6±0.2 | 0.761  0.454  0.875  0.090  0.971 | 4.5±1.0  2.0±1.7  2.6±0.8  1.2±0.2  0.6±0.2 | 4.4±0.9  1.7±1.1  2.6±0.8  1.2±0.3  0.6±0.2 | 0.560  0.174  0.961  0.234  0.965 | 4.8±1.2  1.9±1.3  2.9±1.1  1.1±0.4  0.8±0.1 | 4.9±1.2  2.1±1.4  3.0±0.9  1.0±0.2  0.7±0.3 | 0.988  0.649  0.642  0.375  0.704 |
| UKPDS risk engine | 8.5±3.4 | 9.5±3.1 | 0.012 | 20.5±4.0 | 20.7±3.9 | 0.806 | 39.8±9.7 | 42.8±13.2 | 0.415 |
| Framingham risk score | 3.8±3.9 | 3.9±3.2 | 0.863 | 10.3±5.1 | 10.0±5.2 | 0.641 | 17.9±4.7 | 19.1±5.6 | 0.457 |

IMT, intima medial thickness; BMI, body mass index; DM, diabetes mellitus; CRP, C-reactive protein; LDL-C, low-density lipoprotein cholesterol; HDL, high-density lipoprotein cholesterol; Apo, apolipoprotein; UKPDS, United Kingdom Prospective Diabetes Study.

**Table S2**. Changes in treatment patterns after knowledge of subclinical carotid atherosclerosis results according to UKPDS risk engine score

|  | **United Kingdom Prospective Diabetes Study Risk Engine Score** | | | | | | | | |
| --- | --- | --- | --- | --- | --- | --- | --- | --- | --- |
|  | **Low**  **N=343** | | | **Intermediate**  **N=213** | | | **High**  **N=66** | | |
| Carotid IMT | Negative  N=235 | Positive  N=108 | P-value | Negative  N=101 | Positive  N=112 | P-value | Negative  N=15 | Positive  N=51 | P-value |
| **Achievement of treatment target goals (%)** | | | | | | | | |  |
| BP (<130/80 mmHg) | 116 (50.9) | 50 (47.2) | 0.528 | 47 (48.5) | 51 (49.0) | 0.934 | 3 (21.4) | 18 (39.1) | 0.340 |
| LDL (<2.59 mmol/L) | 84 (68.3) | 45 (72.6) | 0.549 | 39 (68.4) | 38 (67.9) | 0.949 | 5 (62.5) | 20 (72.9) | 0.649 |
| **Treatment pattern (%)** | | | | | | | | |  |
| Changed | 48 (24.1) | 30 (29.1) | 0.346 | 27 (29.7) | 36 (35.3) | 0.406 | 4 (26.7) | 23 (46.0) | 0.183 |
| **Additional medications** | | | | | | | | | |
| Anti-hypertensive drugs | 14 (7.0) | 15 (14.6) | 0.035 | 11 (12.1) | 14 (13.7) | 0.735 | 1 (6.7) | 7 (14.0) | 0.448 |
| Lipid-lowering drugs | 26 (13.1) | 15 (14.6) | 0.719 | 13 (14.3) | 14 (13.7) | 0.911 | 3 (20.0) | 9 (18.0) | 0.861 |
| Antiplatelet agents | 13 (6.5) | 10 (9.7) | 0.324 | 9 (9.9) | 17 (16.7) | 0.169 | 0 (0.0) | 11 (22.0) | 0.055 |
| **Behavior change** **score [Delta(baseline – 6 months)]** | | | | | | | | |  |
| Smoking | -0.01±0.36 | -0.05±0.42 | 0.389 | -0.11±0.51 | -0.03±0.50 | 0.239 | -0.20±0.41 | -0.12±0.44 | 0.547 |
| Drinking | -0.02±0.49 | 0.04±0.34 | 0.232 | 0.04±0.35 | 0.03±0.55 | 0.830 | -0.20±0.56 | -0.04±0.49 | 0.290 |
| Exercise | 0.13±1.37 | 0.18±1.03 | 0.691 | 0.01±1.28 | -0.01±1.54 | 0.923 | -0.20±2.18 | -0.26±1.57 | 0.914 |
| Stress | -0.02±0.67 | -0.05±0.62 | 0.698 | 0.02±0.71 | 0.14±0.62 | 0.175 | 0.00±0.66 | 0.06±0.68 | 0.766 |
| Nutrition (salty food) | 0.03±1.08 | 0.06±1.07 | 0.809 | 0.14±0.87 | 0.00±0.88 | 0.245 | 0.21±0.58 | 0.04±1.09 | 0.426 |
| Drug compliance | -0.08±0.94 | -0.12±0.96 | 0.670 | -0.10±1.12 | -0.05±0.79 | 0.690 | 0.27±1.34 | 0.08±1.35 | 0.637 |

UKPDS, United Kingdom Prospective Diabetes Study; IMT, intima medial thickness; BP, blood pressure; LDL-C, low-density lipoprotein cholesterol.
